# Annotations

**Published:** 1900-02-24

**Authors:** 


### ANNOTATIONS.

### Dust in Camps.

AMONG other good things connoted by the better news from South Africa is that the new distribution of our troops will involve the movement of some of the camps, which have of late been getting distinctly unhealthy. We have been learning at Modder River what the Americans learned in Cuba, that dirty dust and filthy flies are quite as effective as mere pollution of water in distributing the infection. In addition to typhoid fever many of the men at Modder River have been affected with a severe form of diarrhœa of a dysenteric nature, or at least of an inflammatory nature, which is suspected to have been produced by the large amount of sand which the men swallow during the sand-storms which are so prevalent in that region. The sand-storms, or sand-spouts—for really they are of the same nature as water-spouts, except that it is sand instead of water which is drawn up by the eddy of the whirlwind—occur almost every day, and not only are those who have to breathe this sandy atmosphere forced to swallow an enormous quantity of siliceous material, by which the intestinal mucous membrane is irritated, but they have to take in along with it mixed samples of the various organic impurities which go to make up the dust of camps. They have, we understand, sunk wells at Modder River, and the use of the river water for drinking purposes has been put a stop to; but no amount of water sanitation will prevent the spread of typhoid fever in the presence of such dust storms as those described unless the surface of the soil is kept in a more complete state of purity than is at all easy to ensure in a large camp, fixed in one place week after week. There is also a surgical side to this question of dust. In some of the camps, if a dressing is removed even for a moment, this all-pervading dust gets in between it and the wound, and it cannot be doubted that the foulness of this dust in some of the more stationary camps has been the cause of the suppuration which has occurred in some of the most carefully treated wounds. Great as is the strain involved in the movement of an army, life on the march is always healthier than life in a stationary camp.

### The "Procreation of Children."

ACCORDING to the English marriage service matrimony is an honourable estate, ordained, firstly, for the procreation of children, and thus the Church gives its sanction to what has long been one of the striking characteristics of the Anglo-Saxon race, namely, its fecundity, its capacity for producing innumerable children with which to people its wide possessions. Foreigners are apt to mock at the size of English families, and endless are the jokes that have been made at the prolific marriages of our artisans. But it is to these prolific marriages, and to the urgent necessity which they have involved of finding outlets for an exuberant race, that we owe the origin and constant growth of our colonial empire, and the steady spread of the English tongue in every quarter of the globe. Among the many changes which have taken place in England during the past quarter of a century—some good, some bad, some pointing to a higher civilisation, others to a greater selfishness, some to a longer life, others to a lessened stamina—there is one which hardly shows itself to the casual, or even to the careful observer, unless he has access to statistical records, but which reference to the Annual Reports of the Registrar-General makes obvious enough, namely, the lowering of the English birth-rate during recent years, and the lessened number of children in our midst. It appears that the birth-rate for the year 1899 was only 29·26 per 1,000 of the estimated population, as against 36·3 in 1876, the birth-rate of the past year thus being the lowest recorded by the Registrar-General. The year before it was 29·4, and, in fact, it has been steadily declining for a considerable time, and the fall in some of the large towns has been much greater than is expressed by the average for the whole country. What is the origin of this curious declension in the birth-rate it is difficult to say. It can hardly be suggested that it is due to those widespread economic laws in accordance with which the fertility of a race accommodates itself to the means of sustenance available for its use, and we can but fear that it is due to the spread of selfishness and to an unhappy misuse of the fruits of the tree of knowledge. From a purely theoretical point of view it might be maintained that it is better that children should be fewer, the presumption being that the smaller number would be more carefully reared and would thus produce a finer race. Unfortunately we see no sign of such an ideal result. Fertility of race is at the root of many things. Our merchants complain that in every land they find themselves met in the keenest competition by the ubiquitous German. The reason lies with German mothers. They are now producing 36 children a year for every 1,000 of the population, just what English mothers were doing 25 years ago. Now the English mothers are content with 29 per 1,000—one more nail, as some would say, in the coffin of the empire.

### Supine Sanitary Authorities.

AMID all the outcry which we hear raised from time to time for new laws and more stringent legislation in regard to sanitary matters, it is as well to bear in mind to how small an extent the laws which we already possess are put in force by many of the public bodies to whose care the management of all health matters in their respective districts is entrusted. Sir Charles A. Cameron, speaking recently in Dublin upon this subject, said that while the powers of municipal authorities to promote measures for the improvement of the public health were formerly very limited, they had been greatly increased in the latter half of the nineteenth century, and were now very extensive. But although many sanitary authorities used these powers almost to their full extent, and others in a more moderate degree, there were not a few which put them in force only so far as they were compelled to employ them. Many so-called sanitary authorities, he said, have not yet adopted the Notification of Infectious Diseases, the Open Spaces, the Housing of the Working Classes, the Shop Hours, and other useful Acts; had not established abattoirs, baths and washhouses, or disinfecting chambers; nor carried out efficiently the Sale of Food and Drugs, the Margarine, and the Contagious Diseases (Animals) Acts. It would probably be easy to add considerably to this list of neglects and omissions on the part of sanitary authorities, and it is clear that unless the local authorities have the will to carry out sanitary improvements, legislation on the subject has but little real effect in removing the evils aimed at.

